# Acceptance and hesitancy of parents to vaccinate children against coronavirus disease 2019 in Saudi Arabia

**DOI:** 10.1371/journal.pone.0276183

**Published:** 2022-10-18

**Authors:** Ahd Almansour, Sarah M. Hussein, Shatha G. Felemban, Adib W. Mahamid

**Affiliations:** 1 Department of Medical Laboratory Sciences, Fakeeh College for Medical Sciences, Jeddah, Kingdom of Saudi Arabia; 2 Department of Public health, Community Medicine, Environmental and Occupational Medicine, Faculty of Medicine, Suez Canal University, Ismailia, Egypt; King Abdulaziz University Faculty of Medicine, SAUDI ARABIA

## Abstract

**Background:**

Vaccination of masses against coronavirus disease 2019 (COVID-19) is critical to overcome the pandemic and restore normalcy. However, vaccine refusal and hesitancy prevail in many countries. COVID-19 has rapidly spread in Saudi Arabia since 2020. The acceptance rate of COVID-19 vaccines has been investigated in adults aged >18 years in Saudi Arabia. This study aimed to understand the acceptance and hesitancy of parents to vaccinate children aged <12 years against COVID-19 in Saudi Arabia and identify strategies that can encourage their engagement.

**Methods:**

We used an online cross-sectional survey distributed to parents who lived in all regions of Saudi Arabia to investigate parents’ views on the acceptability of a future COVID-19 vaccine for their children aged <12 years. Five hundred parents living in Saudi Arabia completed the survey.

**Results:**

The survey indicated that mothers were more enthusiastic about participating in the study than fathers. The participant aged 37.31 ± 8.52 years. A total of 38.6% of participants refused to vaccinate their children. Additionally, 56% were unsure if the vaccine would cause serious side effects in children. A total of 48.8% of parents believed that the Pfizer vaccine was suitable for children, while 64.5% failed to decide whether to administer vaccines to their children.

**Conclusion:**

Vaccine hesitancy remains a major problem worldwide. A lack of scientific evidence on vaccine efficacy, low education level, and reduced level of health education and promotion are the most common factors in parents in Saudi Arabia. However, some participants agreed to receive vaccines only to protect their family members, and due to governmental rules and school mandates. Therefore, vaccine efficacy and safety in children must be clearly communicated to the public. This information would aid in reducing the hesitancy of parents to vaccinate their children against COVID-19.

## 1. Introduction

The coronavirus disease 2019 (COVID-19) is a pandemic that has impacted millions of people [1]. Additionally, >195 million confirmed COVID-19 cases have been reported globally, including >6 million deaths. Therefore, vaccination of masses against COVID-19 is critical to overcome the pandemic and restore healthy living [2]. However, vaccine refusal and hesitancy are prevalent in many countries, and vaccines are still unavailable for children aged <12 years. Additionally, parents are concerned about children’s well-being, and convincing them for vaccination is a complex process [1].

Vaccine acceptance is complicated and context-specific, varying with the place, time, social class, and community [3–7]. A study in Europe showed that vaccine hesitancy is mainly influenced by safety concerns and adverse effects of vaccination [8]. Another study demonstrated that many healthcare workers in Ireland avoided the seasonal influenza vaccination due to misconceptions and a lack of trust in the vaccine [9, 10].

Since October 17, 2020, COVID-19 has rapidly spread in Saudi Arabia, causing >750,000 cases and 9,000 deaths. The current population of Saudi Arabia is 35,389,457, and >10,000,000 individuals are aged <18 years. A previous study that investigated the rate of acceptance of COVID-19 vaccines observed that only 48% of Saudi adults aged >18 years intended to receive vaccines [2]. These results are also consistent with the total number of Saudi residents who got vaccinated (approximately 50%). However, studies have neither investigated the acceptance rate of COVID-19 vaccines and their determinants in people aged <18 years nor determined the predictors associated with Saudi parents’ intent to vaccinate children when vaccines are available for children aged <12 years. Therefore, this study aimed to understand the acceptance and hesitancy of parents to vaccinate their children against COVID-19 in Saudi Arabia, and identify strategies that can help encourage their engagement.

## 2. Material and methods

### 2.1. Study design

A cross-sectional study was conducted between August 2021 and February 2022.

### 2.2. Study setting and participants

A web-based survey was conducted with parents who lived in all regions of Saudi Arabia, including western, southern, northern, eastern, and central regions.

### 2.3. Inclusion criteria

All parents with children aged <12 years and who used the internet and could read Arabic or English were included in the study.

### 2.4. Exclusion criteria

Other members of the same family.

### 2.5. Sampling and procedure

We performed convenience and snowball sampling. We calculated the sample size by epi-info software version 7. So, a sample size of 385 participants would help achieve a 95% confidence interval (CI) and 5% margin of error. To raise the external validity and generalizability of the study, the sample size was raised to 500 participants. An online survey on Google Forms was distributed to parents in Saudi Arabia through social media platforms including emails, and they were requested to circulate the forms to other parents as well.

### 2.6. Outcome

The rate of acceptance of parents to vaccinate their children.

### 2.7. Data collection tool

The questionnaire was self-administered with both Arabic and English versions to allow participants to choose the appropriate language was conducted. The participants were invited to complete an online survey on Google Forms, along with their ethical consent to participate in the study. The questionnaire included following sections:

#### 2.7.1. Sociodemographic data

Age, sex, employment, marital status, and number and age of child/children.

#### 2.7.2. COVID-19 history

Details of the previous diagnosis of the participant or family members with COVID-19, level of care required, and whether family member succumbed to COVID-19.

#### 2.7.3. Vaccine history of parents

COVID-19 vaccination status, number of doses received, and any adverse effects of vaccines.

#### 2.7.4. Attitude towards COVID-19 vaccines

Opinion on whether vaccines protect children from COVID-19, whether vaccines strengthen the immune system, and whether they would recommend vaccines to other parents.

#### 2.7.5. Acceptance of vaccination of child/children

The parents’ answers were recorded on a 3-points Likert scale as: "disagree, agree, and undecided."

#### 2.7.6. Factors affecting vaccine acceptance

Concerns on vaccinating child/children against COVID-19, safety concerns of the vaccine, source of information on vaccines, concerns on serious reaction to vaccines, whether child/children had serious reaction/reactions to previous vaccinations, and whether parents think that vaccines are suitable for children only if vaccine benefits are larger than their risks, along with easy availability of vaccines.

The questionnaire was adapted from previous studies [11–13]. The original questionnaire in English was bidirectional "back–back" translated into Arabic. Both versions were available for participants. We used the most appropriate and understandable terms and got them revised by three experts. A pilot study was conducted to test the questionnaire on 20 participants to confirm all the language amendments and ease of use and determine the feasibility of the survey. Additionally, we tested the participants’ responses to different items of the questionnaire. The validity was estimated based on whether the questions were comprehensive. Data of the pilot study were excluded from the final analysis.

### 2.8. Statistical analysis

Data entry and statistical analysis were performed using the Statistical Package for Social Science (SPSS) software version 22. Descriptive statistics are presented as tables and graphs. The Student’s *t*-test was used to evaluate quantitative normally distributed variables, and the Mann Whitney U test to evaluate non-normally distributed variables. The Chi-square test was used for qualitative variables. Regression analyses were performed to assess the determinants of acceptance. A p<0.05 was considered to be statistically significant.

## 3. Results

As indicated in Table 1, 77.8% of the participants are mothers, 78.6% are Saudi residents, and 41.4% are residing in Makkah region. The participants’ mean age was 37.31 ± 8.52 years, and 91% were married. A total of 56.6% had a bachelor’s degree, and 56% were working. The mean number of children was 2.84 ± 1.51, and 59.6% of the participants had children aged <12 years.

**Table 1:** Sociodemographic data of the participants (*n* = 500).

Variables	Frequency	%
*Parents*		
Mother	389	77.8
Father	111	22.2
*Nationality*		
Saudi	393	78.6
Non-Saudi	107	21.4
*Age of parents*	37.31 ± 8.52 years	
*Marital status*		
Married	455	91.0
Divorced	35	7.0
Widow	10	2.0
*Residence in KSA*		
Makkah region	207	41.4
Medina region	68	13.6
Riyadh region	38	7.6
Eastern region	36	7.2
Ha’il region	20	4.0
Al-Qassim region	26	5.2
Jizan region	26	5.2
Asir region	23	4.6
Algof region	56	11.2
*Educational level*		
High school	63	12.6
Bachelor’s	283	56.6
Postgraduate	129	25.8
Others	25	5.0
*Employment*		
No	220	44.0
Yes	280	56.0
*Number of children*	2.84±1.51	
*Age of children*		
<12 years	298	59.6
12–18 years	56	11.2
Both	146	29.2

KSA, Kingdom of Saudi Arabia.

**Fig 1:**
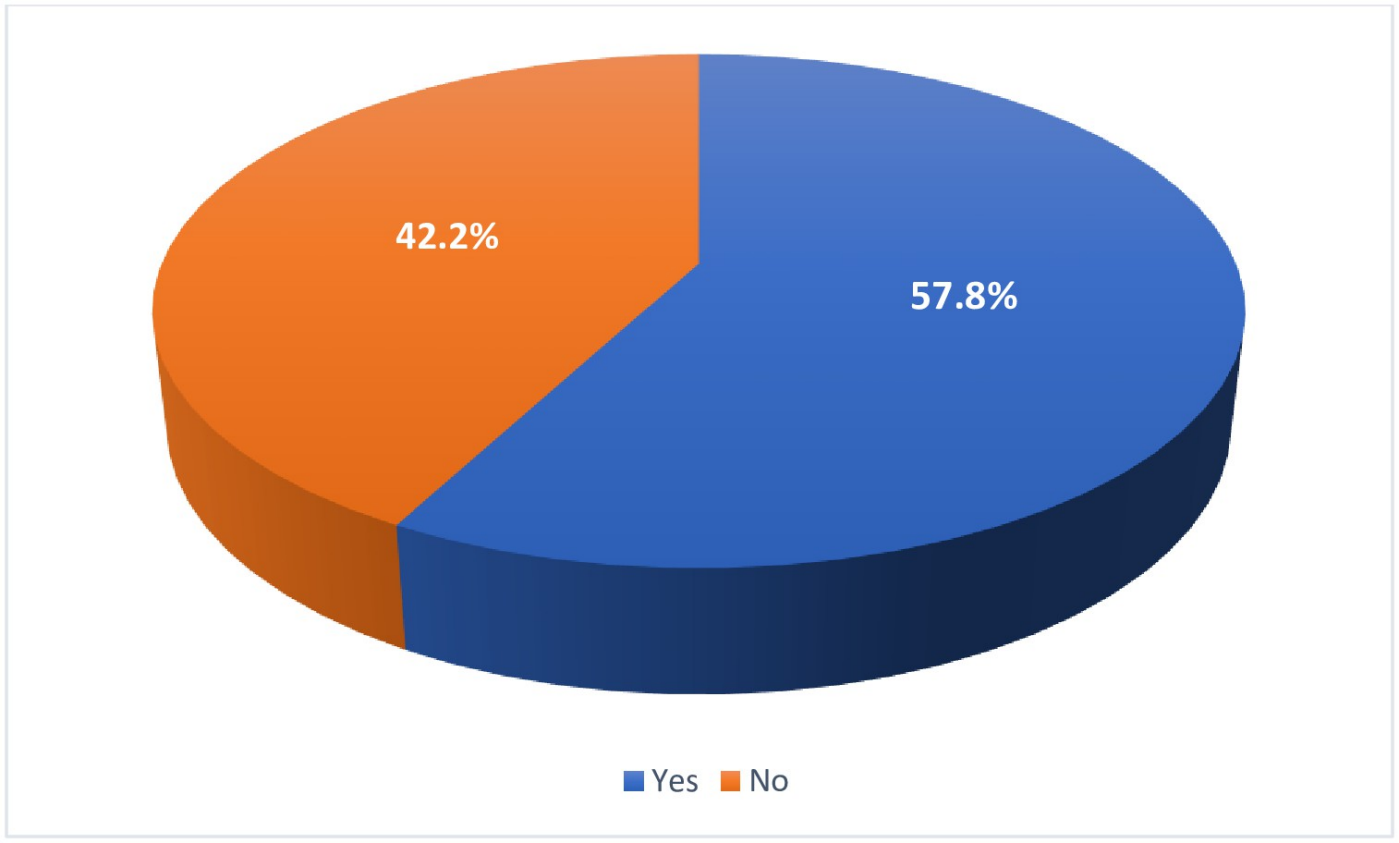
Frequency distribution of participants who have/would vaccinate child/ren against COVID-19 (*n* = 500).

As shown in Fig 1, 289 participants (57.8%) have or would vaccinate their children against COVID-19, and 211 participants (42.2%) have not or would not vaccinate their children against COVID-19.

**Fig 2:**
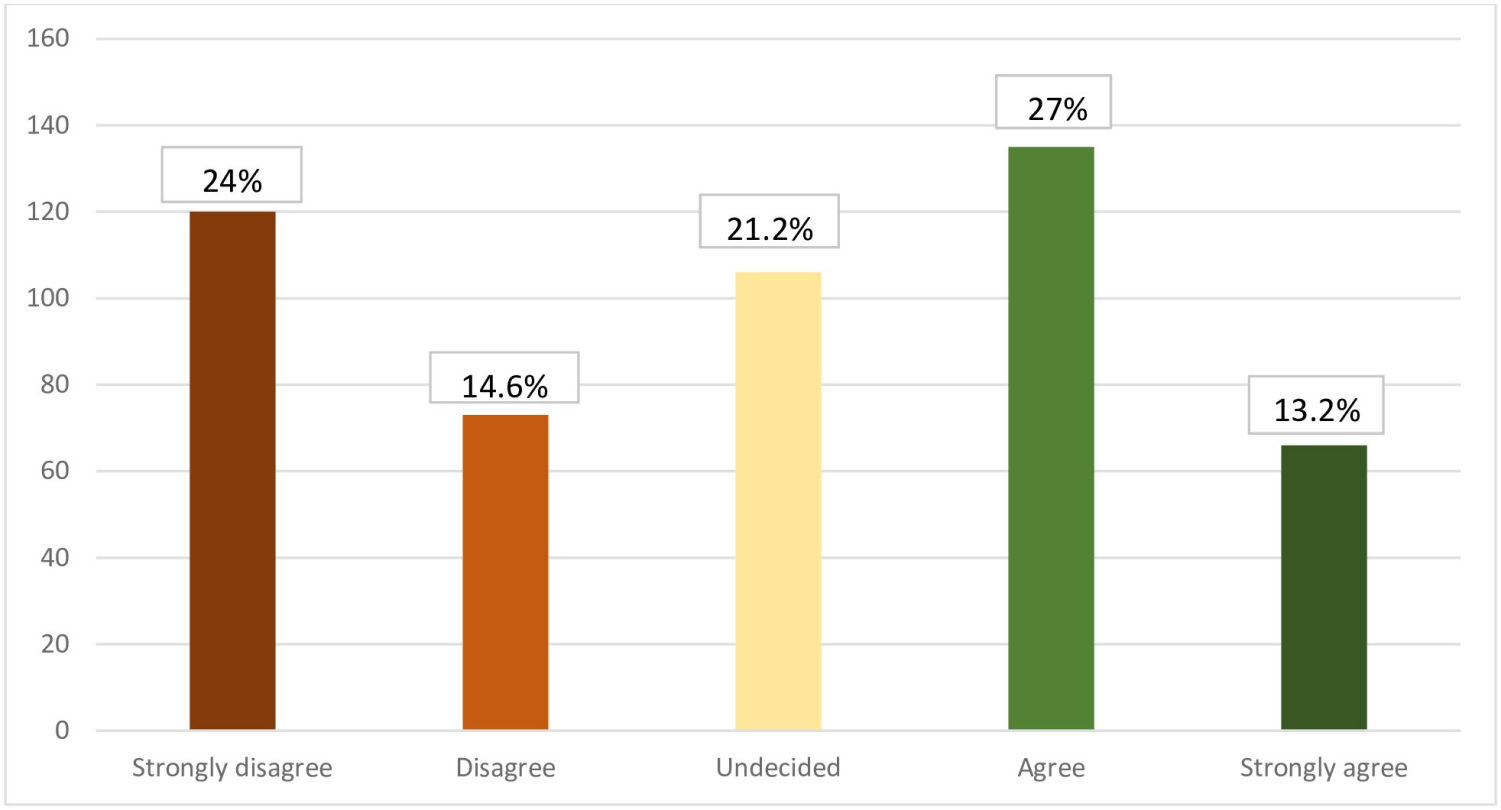
Frequency distribution of acceptance pf parents to vaccinate their children against COVID-19 (*n* = 500).

Regarding acceptance on vaccinating children against COVID-19, as displayed in Fig 2, 13.2% of the participants strongly accept, while 24% strongly disagree on vaccinating their children.

We analyzed the attitude of participants towards vaccines. As indicated in Table 2, 29.6% of the participants are concerned about their children acquiring COVID-19, 57.8% believe that vaccines would strengthen the immune system, 50.2% believe that vaccine benefits are greater than its risk, and only 36% believe that vaccines would protect children from COVID-19. The most common reason for vaccinating the children was the school mandate. A total of 38.4% of the participants believe that COVID-19 vaccines would be moderately safe for children. However, 56% were unsure on whether vaccines would cause serious side effects in children.

**Table 2:** Attitude of participants towards vaccines (*n* = 500).

Variables	Frequency	%
*How much concerned are you about your child/ren acquiring COVID-19*?		
Not concerned at all	48	9.6
Not concerned	104	20.8
Undecided	61	12.2
Concerned	139	27.8
Very concerned	148	29.6
*Do you think that vaccines would strengthen the immune system*?		
No	92	18.4
Yes	289	57.8
Not sure	119	23.8
*Do you think that vaccine benefits are greater than its risk*?		
No	117	23.4
Yes	251	50.2
Not sure	132	26.4
*Do you think that vaccines would protect children from COVID-19*?		
No	169	33.8
Yes	180	36.0
Not sure	151	30.2
*Why did/would you vaccinate your children*?		
Became obligatory	122	24.4
Required for school attendance	166	33.2
Convinced by its importance	66	13.2
All these causes	146	29.2
*The most common source of information about vaccines*?		
News on television/ national radio	81	16.2
Government agencies	90	18.0
Social media	140	28.0
Discussion with friends and family	30	6.0
Healthcare providers	159	31.8
*Do you think COVID-19 vaccines are safe for children*?		
Not safe at all	132	26.4
A little safe	98	19.6
Moderately safe	192	38.4
Very safe	78	15.6
*Do you think that vaccines would cause serious side effects in children*?		
No	52	10.4
Yes	168	33.6
Not sure	280	56.0
*Which vaccine do you think is suitable for children*?		
AstraZeneca	19	3.8
Pfizer	244	48.8
Moderna	10	2.0
Undecided	227	45.4
*Do you think that vaccines would be easily available*?		
No	54	10.8
Yes	446	89.2
*Would you recommend vaccines to others with children*?		
No	256	51.2
Yes	244	48.8

COVID-19, coronavirus disease 2019.

Further, healthcare workers provided information on COVID-19 vaccines to 31.8% of the participants (Table 2). Additionally, 48.8% of the parents believed that Pfizer vaccine was suitable for children; but, 51.2% do not believe that vaccines would be available easily. Furthermore, 48.8% of the participants would recommend others to vaccinate their children.

A total of 45.4% of the participants or someone in their family were previously diagnosed with COVID-19 (Table 3). Among them, 23.4% did not require any medical support, while 86% lost a family member due to COVID-19.

**Table 3:** History of COVID-19 in participants (*n* = 500).

Factors affecting COVID-19 vaccination in children	Frequency	%
*Were you or someone in the family previously diagnosed with COVID-19*?		
No	267	53.4
Yes	227	45.4
Not sure	6	1.2
*If yes*, *what kind of medical support was required*? *(n = 2327)*		
Medical support not required	117	23.4
Required medical support without hospitalization	99	19.8
Required hospitalization	17	3.4
*Did you lose a family member due to COVID-19*?		
No	430	86.0
Yes	60	12.0
Not sure	10	2.0
*Are you aware of any child with a serious reaction due to vaccination that needed medical support*?		
No	413	82.6
Yes	87	17.4

COVID-19, coronavirus disease 2019.

As indicated in Table 4, factors that influenced COVID-19 vaccination in children were as follows: (a) being mother: 71.3% of the participants who vaccinated their children were mothers; (b) the mean of age of participants who vaccinated (39.10 ± 9.714 years) children was higher than those who did not (34.86 ± 6.839) vaccinate their children; (c) marital status: 89.3% of the participants who vaccinated children were married; (d) educational level: the most common education in the two groups was bachelor’s degree; and (e) employment: 60.2% of the participants who vaccinated children were working. Moreover, the number of children and their ages were significant factors that affected vaccination of children. Awareness of any child with serious reaction due to vaccine that required medical aid was significantly lower in parents who vaccinated (12.1%) than in those who did not vaccinate (24.6%) their children. Further, 62.6% of the participants who vaccinated their children believed that the Pfizer vaccine was the most suitable for children, while 64.5% of the participants who did not vaccinate their children could not decide on the suitability of vaccines. A higher proportion of participants (94.1%) who vaccinated children believed that vaccines would be easily available than those who did not vaccinate children (82.5%) (p<0.05).

**Table 4:** Factors affecting vaccination of children against COVID-19 (*n* = 500).

Factors	Vaccination status				p value
	No (*n* = 211)		Yes (*n* = 289)		
	No.	%	No.	%	
*Parents*					<0.001*
Mother	183	86.7	206	71.3	
Father	28	13.3	83	28.7	
*Age of participants (years)*	34.86 ± 6.84		39.10 ± 9.71		<0.001*
*Marital status*					0.028*
Married	197	93.4	258	89.3	
Divorced	13	6.2	22	7.6	
Widow	1	0.5	9	3.1	
*Educational level*					0.027*
High school	19	9.0	44	15.2	
Bachelor’s	120	56.9	163	56.4	
Postgraduate	60	28.4	69	23.9	
Others	12	5.7	13	4.5	
*Nationality*					>0.999
Saudi	166	78.7	227	78.5	
Non-Saudi	45	21.3	62	21.5	
*Employment*					0.029*
No	105	49.8	115	39.8	
Yes	106	50.2	174	60.2	
*Number of children*	2.46 ± 21.27		3.12 ± 1.61		<0.001*
*Age of children*					
<12 years	172	81.5	126	43.6	
12–18 years	5	2.4	51	17.6	<0.001*
Both	34	16.1	112	38.8	
*The participant or someone in family previously diagnosed with COVID-19*?					0.067
No	102	48.3	165	57.1	
Yes	108	51.2	119	41.2	
Not sure	1	0.5	5	1.7	
*Lost a family member due to COVID-19*?					0.267
No	179	84.8	251	86.9	
Yes	27	12.8	33	11.4	
Not sure	5	2.4	5	1.7	
*Most common source of information about vaccines*					0.031*
News on television/national radio	32	15.2	49	17.0	
Government agencies	37	17.5	53	18.3	
Social media	47	22.3	93	32.2	
Discussion with friends and family	18	8.5	12	4.2	
Healthcare providers	77	36.5	82	28.4	
*Awareness on any child with a serious reaction due to COVID-19 vaccine that required medical assistance*?					<0.001*
Yes	52	24.6	35	12.1	
No	159	75.4	254	87.9	
*Vaccine that would be suitable for children*					
AstraZeneca	8	3.8	11	3.8	
Pfizer	63	29.9	181	62.6	<0.001*
Moderna	4	1.9	6	2.1	
Undecided	136	64.5	91	31.5	
*Would vaccines be available easily*?					<0.001*
No	37	17.5	17	5.9	
Yes	174	82.5	272	94.1	

* *p*<0.05; COVID-19, coronavirus disease 2019.

Next, we performed logistic regression analysis of predictors that influenced COVID-19 vaccination of children. The significant predictors were age of parents (odds ratio [OR], 1.063; 95% CI, 1.063–1.114; p = 0.012), educational level (OR, 0.627; 95% CI, 0.406–0.968; p = 0.035), age of children (OR, 2.855; 95% CI, 1.896–4.299; p<0.001), belief that COVID-19 vaccines were safe for children (OR, 2.464; 95% CI, 1.598–3.800; p<0.001), acceptance of COVID-19 vaccines (OR, 3.684; 95% CI, 2.583–5.253; p<0.001), and type of COVID-19 vaccines suitable for children (OR, 0.696; 95% CI, 0.517–0.938; p = 0.017) (Table 5).

**Table 5:** Multivariate logistic regression analysis of factors affecting decision of vaccinating children against COVID-19 (*n* = 500).

Factors	B	OR	p value	95% CI for OR	
				Lower	Upper
Age of parents	0.061	1.063	0.012*	1.013	1.114
Parents (Mother)	-0.383	0.682	0.342	0.310	1.502
Marital status	0.297	1.346	0.487	0. 583	3.110
Educational level	-0.467	0.627	0.035*	0.406	0.968
Employment (No)	-0.369	0.691	0.264	0.362	1.321
Number of children	0.059	1.061	0.655	0.819	1.375
Age of children	1.049	2.855	<0.001*	1.896	4.299
Belief that vaccines would strengthen the immune system	-0.094	0.809	0.411	0.488	1.341
Belief that vaccine benefits are greater than its risk	-0.254	0.776	0.304	0.478	1.259
Belief that vaccines would protect children from COVID-19	0.089	1.093	0.669	0.727	1.645
Belief that COVID-19 vaccines are safe for children	0.902	2.464	0.000*	1.598	3.800
Belief about serious side effects of vaccines in children	0.278	1.320	0.326	0.758	2.299
Acceptance of COVID-19 vaccines for child/ren (No)	1.304	3.684	<0.001*	2.583	5.253
Concern about child/ren acquiring COVID-19	-0.207	0.813	0.090	0.640	1.033
Awareness of any child with serious reaction due to vaccines that required medical assistance	-0.504	0.604	0.251	0.255	1.429
Type of COVID-19 vaccine suitable for children	-0.362	0.696	0.017*	0.517	0.938
Belief about easy availability of vaccines for children (No)	-0.070	0.932	0.885	0.361	2.407

COVID-19, coronavirus disease 2019; OR, odds ratio; CI, confidence interval;

* *p*<0.05; Nagelkerke R Square = 0.719.

## 4. Discussion

COVID-19, caused by severe acute respiratory syndrome coronavirus (SARS-CoV-2), is an emerging pandemic since its first clinical observation in December 2019 in Wuhan, China [13]. The pandemic has led to loss of life in many age groups along with extensive family disruption and social distancing [15]. With limited therapeutic options for COVID-19, vaccine development has been boosted worldwide [1]. COVID-19 vaccines are major public healthcare achievement of the 21^st^ century, and vaccines have apparently decreased the disease severity and progression [15]. Nevertheless, the success of COVID-19 vaccines depends on achieving herd immunity.

People’s unwillingness to receive vaccines has been categorized as “vaccine hesitancy.” Several studies have studied the causes of such hesitancy in various populations. The present survey focused on the rate of acceptance of COVID-19 vaccines in 500 parents from different families in Saudi Arabia. We compared the results of the present study with that of a Turkish study comprising 428 participants and an English study comprising 1,252 participants [11, 13]. The mean age of participants in all the three studies ranged between 30 and 40 years [11, 13]. The present study found that mothers were significantly more enthusiastic for participating in the study than fathers. This result is similar to that reported by Yigit et al. Moreover, there was no significant difference in vaccine compliance in participants from different geographical locations.

Bell et al. observed that approximately 90% of the parents accepted vaccinating children against COVID-19, while Goldman et al. reported a 65% acceptance rate [14]. The main reasons for this acceptance were protection of children from COVID-19 complications and protection of families who were at high risk of acquiring COVID-19. In the current study, 38.6% of the parents denied vaccinating their children, but >50% of the participants agreed to vaccinate children. The findings suggest that the most common motive for vaccinating the children was school attendance. Less than 40% of the participants believed that vaccines were protective. Nevertheless, 89% of the parents believed that vaccines were acceptable, yet <50% were hesitant to vaccinate their children. In the study by Yigit et al., the most important reason for vaccine compliance was the willingness to protect patients with chronic diseases living together in the same house [13]. The motive in the present study was similar with a major factor for vaccinations was protecting a family member from another relative diagnosed with COVID-19.

Vaccine hesitancy is the most significant barrier to vaccine acceptance worldwide. The hesitancy is linked to certain factors, such as vaccination history, side effects, safety and efficacy, lack of confidence in the healthcare system, and whether the government provided vaccine gratuitously [15]. Some participants (<50%) in the present study believed that vaccines may not strengthen the immune system. This could be attributed to a lack of knowledge on vaccine importance in the parents or lack of scientific evidence on the efficacy of the vaccines. Nevertheless, some parents (50%) believed that certain types of vaccines were more beneficial and had greater benefits than risks. This finding explains the preferential selection of the Pfizer vaccine by parents in the present study. The study by Bell et al. suggested that the main reason for vaccine hesitancy was insufficiency of scientific evidence to support vaccination. However, 62% of the participants were concerned about vaccine safety [11]. Therefore, educating the population with scientific evidence would convince them on the vaccines and reduce uncertainty. Additionally, parents may tend to be more protective about their children than themselves. Thus, the parents’ decision to vaccinate children differs from that for vaccinating adults [1].

Yigit et al. observed that the rate of rejection of domestic vaccines increased with an increase in education level [13]. In the current study, approximately 56% of the parents were educated; and, 86% lost a family member, that inspired them to undergo vaccination. Therefore, the education level is the most common factor influencing the parental decision on children’s vaccination. Yigit et al. suggested that the side effects of vaccines was the main reason for the unwillingness of participants to vaccinate their family against COVID-19, while 9% of participants failed to believe in the effectiveness of vaccines. Moreover, <1% believed that vaccines may contain cellular microchips [13]. This false belief emphasizes the lack of scientific knowledge about the vaccine’s effects on the body. The study by Bell et al. indicated that 3% of the participants vaccinated their children only to avoid social distancing and attain normal lives [11]. The remaining participants are vaccine hesitant. These results are slightly different from those of the present study that suggested that the main reason for vaccine hesitancy was the lack of scientific evidence on vaccine efficacy. Goldman et al. highlighted similar reasons for vaccine hesitancy—the questionable novelty of the vaccine [1].

The study had inherent limitations, as the study used an electronic survey platform which was useful for data collection, this limited the study’s ability to define and describe the population. In addition, the sample size was convenient with biased respondents.

In conclusion, vaccine hesitancy remains a major problem worldwide. Therefore, it is crucial to understand and address factors that may affect COVID-19 vaccine acceptability in parents to prevent vaccine inequalities. The most common factors were a lack of scientific evidence on vaccine efficacy, low education level, and reduced level of health education and promotion. However, some populations undergo vaccination only to protect their family members, due to governmental rules, and for school attendance. Additionally, information on different approaches for COVID-19 vaccination, including vaccine efficacy and safety in children, must be clearly communicated to the public. This information would aid in reducing the hesitancy of parents to vaccinate their children against COVID-19.

## Supporting information

S1 File (SAV)
