# Correction: Acceptance and hesitancy of parents to vaccinate children against coronavirus disease 2019 in Saudi Arabia

**DOI:** 10.1371/journal.pone.0307674

**Published:** 2024-07-18

**Authors:** Ahd A. Mansour, Sarah M. Hussein, Shatha G. Felemban, Adib W. Mahamid

The first author’s name is spelled incorrectly. The correct name is: Ahd A. Mansour. The correct citation is Mansour AA, Hussein SM, Felemban SG, Mahamid AW (2022) Acceptance and hesitancy of parents to vaccinate children against coronavirus disease 2019 in Saudi Arabia. PLOS ONE 17(10): e0276183. https://doi.org/10.1371/journal.pone.0276183.
